# Endogenous Development Models and Paths Selection of Rural Revitalization from the Perspective of Ecological Environment Advantages: A Case Study of Nanshi Village, China

**DOI:** 10.3390/ijerph191911979

**Published:** 2022-09-22

**Authors:** Xinwei Guo, Bin Yu, Meiyan Yan, Hui Guo, Junhu Ren, Hanxia Zhang, Zonggang Zhang

**Affiliations:** 1 Key Laboratory for Geographical Process Analysis & Simulation of Hubei Province, Central China Normal University, Wuhan 430079, China; 2 College of Urban and Environmental Sciences, Central China Normal University, Wuhan 430079, China; 3 College of Teacher Education, Dali University, Dali 671003, China

**Keywords:** rural revitalization, ecological economy, ecological products, ecological industry, “two mountains” theory

## Abstract

This article aims to discuss how to give full play to the comparative advantages of the rural ecological environment and realize the endogenous development of rural society and economy in China. First, based on the ecological economy theory of “lucid waters and lush mountains are golden and silver mountains” (the “two mountains” theory), we integrated the theories and methods of ecology, economics, and geography disciplines to examine the transformation of “ecological advantages” into “economic development” from a comprehensive perspective. Second, based on the matching relationship between the division of major function zones and the classification of ecological services, we creatively constructed a theoretical framework for the endogenous development of rural areas. Third, accounting indicators and methods for rural ecological products’ biophysical quantity and monetary value are established. Finally, we conducted an empirical study of Nanshi Village in central China as a case. The results showed that: The benefits provided by ecosystems to the development of human society would be underestimated if it is measured only by the provisioning services; the per capita Gross Ecosystem Product (GEP) of the case area was three times the per capita disposable income of rural permanent residents in the same period. Taking advantage of the rural ecological environment to promote the actual transformation of the potential value of ecological products is the feasible path for rural revitalization. One of the implications of this study is that it links the rural ecological and environmental advantages with social and economic development from the perspective of ecological economics and provides decision-making support for this case and other similar rural ecological industry revitalization practices.

## 1. Introduction

Giving full play to the comparative advantages of the rural areas and exploring the endogenous development models and paths of rural areas according to local conditions has vital theoretical significance and practical value [1]. Rural areas are distinguished from cities in terms of the natural environment and agricultural production (cities belong to a kind of humanistic environment, mainly for non-agricultural production), and the richness of ecological products are incomparable advantages of rural areas over cities. Transforming the advantages of the ecological environment into the benefits of social and economic development is a beneficial exploration to realize rural endogenous growth, which can significantly help rural revitalization [2,3]. Developing countries have a large number of impoverished villages with rich ecological resources, and these areas often shoulder the dual task of protecting the environment and developing the economy. Taking China, the world’s largest developing country, as an example, the 14 concentrated poverty-stricken areas designated by the government overlap highly with 25 national key ecological function areas. Thus, how the ecological and environmental advantages can be transformed into social and economic development advantages has become an essential issue of common concern for government departments and academia.

The ecological economy contains two layers of connotation: Ecological environment and economic development [4]. Green development and ecological and environmental issues have always been hot spots of academic attention [5,6]. International research on ecological and environmental issues has experienced a cultural shift from focusing on ecological and environmental issues to the social-ecological system [7]. The research content has shifted from the quantitative measurement of ecosystem stability and quality to studying the relationship between human health, equity, and well-being under the influence of the natural environment [8,9,10,11]. The effects of the ecological environment on the local society and economy have further expanded to the concern about global food security and poverty alleviation in the vast number of developing countries [12]. The challenge of addressing ecosystem issues has also shifted from resource-intensive ecosystem function restoration to multi-pronged social-ecological system restoration [5,13,14]. It has promoted the regional development policy with the goal of sustainability to rise to the global strategy under the leadership of the United Nations [15]. Rural development and spatial poverty are persistent global challenges, and rural revitalization is a common pursuit of governments of all countries [2,16,17]. Modern China has experienced a century of exploration in rural revitalization. International rural revitalization practices have also provided many experiences for reference [18,19]. Due to the differences in national conditions, systems, and development stages, global rural development strategies can inspire China but cannot replicate the reality of China. China, which has a vast rural area, a large rural population, and is still a developing country, must explore a more suitable rural development strategy [20]. In 2017, the Chinese government proposed the rural revitalization strategy, and the Chinese academic community actively responded to the demand to serve the national strategy [21]. A series of studies on the scientific connotation, evaluation index, development model, and realization path of rural revitalization have been carried out from multiple angles, including sociology, economics, geography, management, and others [22,23,24]. The study found that cultivating endogenous power based on one’s advantages is a common choice to achieve sustainable rural development.

The interaction between the ecological environment and regional development has gradually become a topic of concern among governments and scholars worldwide [25,26]. Social and economic growth is subject to the necessary constraint of the carrying capacity of the natural ecological environment because the complex interaction of the social-economic and ecological-environmental drives the formation of poverty traps. Ecological and poverty problems often overlap in rural areas [5]. As a comprehensive proposition, the interaction between ecological environment and regional development has attracted the participation of natural sciences and humanities and social sciences scholars. In terms of research content, natural sciences scholars have focused on the products and services provided by the ecosystem for human socioeconomic development and have conducted a series of studies around the definition of the concept, construction of evaluation indicators, improvement of accounting methods, and value estimation of ecosystem services [27,28,29,30,31]. Humanities and social scholars are profoundly aware that regional social and economic development should be constrained by the carrying capacity of the natural ecological environment [32,33]. Under the guidance of the United Nations Sustainable Development Framework, they have extensively focused on rural poverty alleviation research on the effects of vulnerable environments in many developing countries [15,34]. In terms of research methods, the accounting of the economic value of ecosystem services currently involves two approaches: One is the economic method based on a consumer perspective, that is, the financial method based on human welfare, which uses money to quantify the services provided by ecosystems to humans [29,30,35,36]. The second is the ecological method based on the perspective of the ecosystem supply, that is, based on the principle of ecological thermodynamics, the energy monetary value of the ecosystem is calculated through the energy currency ratio [37,38]. The former uses the money to directly represent the value of ecosystem services, which has strong operability and broad applicability and has been widely used in research [30]. To sum up, the interaction between environmental protection and regional economic development has increasingly attracted the attention of the academic community [25,29,39,40]. People’s understanding of the relationship between environmental protection and regional economic growth has also changed from intensified production to sustainable intensification [41,42], and rich achievements have been achieved. However, the discussion on how to use the value transformation of ecological products to achieve rural economic development remains insufficient.

Ecological products are in line with ecosystem services, which refer to the final products and services provided by ecosystems for human well-being through biological production and its interaction with human labor [43]. The value realization of ecological products is the process of promoting the transformation of ecological factors into production factors and resource endowments into material wealth through socialized production and market-oriented management. The countryside is an area rich in ecological resources. By valuing ecological products, providing more abundant and higher-value ecological products and services is the preferred path to realize rural revitalization using its advantages. Focusing on the theme of the relationship between ecological products and rural revitalization, most existing studies have been conducted from the perspectives of political economy and social management [44,45]. In the case of limited resources, studies that combine the qualitative and quantitative factors to determine the priority of rural ecological products’ value transformation have been scarce. This paper offers new ideas for solving this problem.

In summary, existing studies still have the following shortcomings: (1) The integration between natural sciences and humanities sciences remains lacking. The natural sciences research focuses on improving the accounting methods of the ecological service value at different regional scales, and the content focuses mainly on the quantitative measurement of the changes in the value and structure of the ecological services. The humanities and social sciences focus on the effects of ecological and environmental damage on regional social and economic development and people’s health and well-being. Most of them regard the ecological environment and changes as a background of economic and social development and prioritize qualitative analysis. The study on the two-way transformation between ecological environment protection and social and economic growth needs to be deepened [30,39,43,44]. (2) In terms of specific methods, the value realization of ecological products is still at the stage of exploration and practice. On the one hand, the realistic transformation system and mechanism of the potential value of the ecological environment are not yet sound. On the other hand, no unified value accounting for the indicators and methods of the products and services provided by the ecological environment exists, and thus, the foundation for the transformation of the value of ecological products is also relatively weak [29,30,36,46,47,48]. (3) The differences in the positioning of regional functions and the comparative advantages of local ecology are often ignored in the specific practice. The selection of regional ecological and economic development models tends to be homogeneous, which weakens the guiding role of research in local practice. In selected cases, the two-way transformation of ecological-environmental and social-economic development advantages can be found mainly in developed countries [1,49]. The case exploration of how to give play to the ecological and environmental benefits of achieving rural social and economic development in developing countries is still insufficient.

The innovation and value of this paper are as follows: First, our study integrates multi-disciplinary theories and methods such as ecology, economics, geography, and planning. It examines the complex proposition of the two-way transformation of “ecological-environmental advantage” and “social-economic development” from a comprehensive perspective. The second is to build an analysis framework for the endogenous development of rural revitalization to realize the value of ecological products and sort out and integrate the value accounting methods of ecological products suitable for rural areas and provide essential support for implementing the value of the ecological products. The third is to take the ecological economy practice of a village in China, which is abundant in ecological resources and backward in the social economy, as an example to carry out research, which has general reference value for the development of villages with comparative ecological advantages in developing countries.

The specific objectives of this study are as follows: (1) To construct a theoretical framework for the endogenous development of rural areas by exploring the advantages of the ecological environment and providing a reference for similar villages. (2) To establish the accounting indicators and methods of biophysical quantity and monetary value of rural ecological products to provide a scientific basis for the value transformation of ecological products. (3) Based on the theoretical construction and methodological modeling, this paper takes Nanshi Village in China as an example, which has the dual tasks of ecological environment protection and rural development, carrying out an empirical study by combining qualitative and quantitative methods.

## 2. Research Framework and Case Selection

### 2.1. Research Framework

(1) Taking the “two mountains” theory as guidance for development. The “two mountains” theory is the guiding theory of ecological economy development proposed by the Chinese government in the new era. In the theory of “two mountains,” “lucid waters and lush mountains” refer to the high-quality ecological products that exist objectively in nature, and “golden and silver mountains” refer to the abundant social wealth created by human labor. “Lucid waters and lush mountains are invaluable assets” is the core value concept of the “two mountains” theory, meaning that high-quality ecological products can not only directly meet people’s growing needs for a better environment but also meet people’s needs for a better life through ecological value transformation and economic value appreciation [29,45,48]. Rural areas have an excellent natural environment and abundant ecological products. The value realization of the ecological products (realization of natural resource assets) is a valuable resource for rural endogenous development and a preferred path for rural ecological industrial revitalization.

(2) Taking the Major Function Oriented Zoning (MFOZ) as the constraint condition of development. MFOZ is the blueprint for China’s territory’s future development and protection pattern [50,51]. MFOZ refers to the function and role performed by a specific region in the natural resources and ecosystems, human social production activities, and life activities in a larger spatial scope. MFOZ is the regional division and major function positioning based on the carrying capacity of resources and environment, the existing development intensity, and future development potential. According to the development content, it can be divided into three types: Urbanized zones, main agricultural production zones, and key ecological function zones in China [52]. The zoning and positioning of major functions are the inherent requirements for the region’s green, coordinated, and sustainable development. Rural revitalization must be compatible with a regional primary function strategy [53]. MFOZ is the constraint condition of rural revival and the value transformation of its ecological products.

(3) Taking the value realization of ecological products as the main line. Ecological products refer to various services that natural ecosystems provide to human society, including provisioning services, regulating services, and cultural services [29,54]. The advantages of ecological services can be determined according to the proportion of each ecological service value in the total value of ecological services, thereby classifying villages into three types: Regulating services dominant, provisioning services dominant, and cultural services dominant, which respectively indicate that regulating services value, provisioning services value, and cultural services value occupy the largest proportion in the value structure of rural ecological products, thus occupying a dominant position. Accordingly, a basic correspondence between the division of major function zones and the classification of ecological services exists: Key ecological functional zones focus on providing ecological regulating services, main agricultural production zones focus on providing provisioning services, and urbanized zones focus on providing non-agricultural production services (such as cultural services) [52], thereby constructing the relationship matrix between the division of major function zones and the classification of ecological services (Table 1). Two types and nine categories of matching relationships are generated. The first type includes three combinations of the diagonal of the matrix, which maintain a corresponding fundamental relationship between the division of major function zones and the classification of ecological services, called the coordinated type. The second type contains six varieties on both sides of the diagonal of the matrix, which misses the corresponding fundamental relationship between the two, and is called the uncoordinated type. The matching relationship is the scientific basis for realizing the value of ecological products and deconstructing the rural revitalization models.

In addition, the value realization of ecological products has three basic paradigms based on how natural resource assets are realized in China.

① Ecological productization. Natural resource assets (ecological product functions) are realized directly through relevant ecological equity transactions, and the external manifestation is the transformation of ecological value, for example, carbon emission rights trading and various ecological compensation.

② Industrial ecologization. Natural resource assets are realized indirectly through the internalization of ecological environment quality into the quality of relevant products, and the external manifestation is the appreciation of economic value, for example, various ecological products and geographical indication products.

③ Ecological industrialization. Natural resource assets are realized comprehensively through the derivation of ecological and cultural landscapes into cultural service products, and the external performance is the transformation of comprehensive value, for example, rural scenery tourism and ecological shopping.

**Figure 1:**
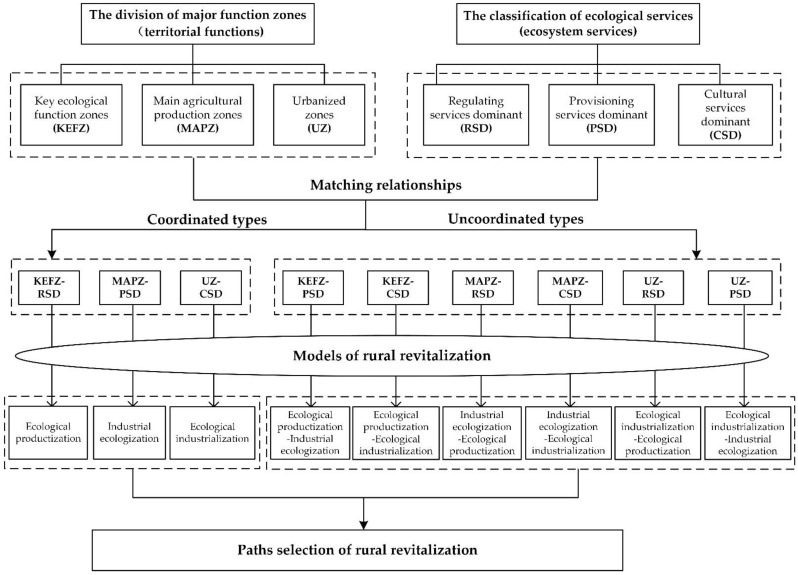
Research Framework.

Accordingly, the endogenous development models and paths of rural revitalization dominated by the value realization of ecological products are clarified (Table 2). The numbers in Table 2 represent the same connotation as Table 1. The research framework of the study is shown in Figure 1.

### 2.2. Research Methods

According to Section 2.1 and Table 1, the scientific accounting of ecological products’ value is the essential support for realizing the value of ecological products.

In this study, we calculated GEP mainly based on the IPBES ecosystem services classification [55] and the System of Environmental-Economic Accounting 2012: Central Framework (SEEA) issued by the United Nations Statistics Division [56]. We have taken Chinese national standards (draft for comments) [57] and the technical guideline on GEP issued by the Chinese Academy of Environmental Planning [58] as the primary reference. Besides, we also drew on previous research results and domestic pilot examples [28,29,30,54,59,60,61], combined with the actual natural resource assets in rural areas. Eventually, we constructed an ecological products value accounting index system in rural areas that included three major types: Provisioning services, regulating services, and cultural services. Among them, provisioning services include five items of agricultural crop, forestry, animal husbandry, fishery, and other production. Regulating services include ten items of water connotation, flood mitigation, carbon sequestration, oxygen release, windbreak and sand fixation, soil erosion prevention, climate regulation, air purification, water purification, and biodiversity maintenance. Cultural services correspond to ecotourism. Our work applies spatially explicit integrated ecological–economic modeling that calculates the biophysical quantity of ecosystem services and then uses economic valuation methods to estimate the monetary value of ecosystem services. The specific accounting index system and methods are shown in Table 3.

**Table 3:** A framework for accounting for the value of ecological products (natural resource assets) in rural areas.

Types of Ecosystem Services	Category of Ecosystem Services	Indicators of Biophysical Quantity	Biophysical Quantity Calculation Methods	Indicators of Monetary Value	Monetary Value Calculation Methods
Provisioning services	agricultural crop products	Production of agricultural crop products	$\mathrm{Method}\;\mathrm{of}\;\mathrm{statistical}\;\mathrm{survey}\;E_{p}=\overset{n}{\underset{i=1}{\sum }}E_{i}$ *E_p_* is the biophysical quantity of products supplied by natural resources, *E_i_* is the output of products supplied by natural resources *i*, *n* is the total number of natural resource products involved in accounting	Monetary value of agricultural crop products	$\mathrm{Method}\;\mathrm{of}\;\mathrm{market}\;\mathrm{value}\;V_{m}=\overset{n}{\underset{i=1}{\sum }}E_{i}*P_{i}$ *V_m_* is the total monetary value of the supplied products, *P_i_* is the unit price of the supplied products of category *i*
	forestry products	Production of forestry products		Monetary value of forest products	
	animal husbandry products	Production of animal husbandry products		Monetary value of animal husbandry products	
	fishery products	Production of fishery products		Monetary value of fishery products	
	other products	Production of other products		Monetary value of other products	
Regulating services	water connotation	Amount of water conservation	Method of water balance [29,54,57,58,59] $Q_{wr}=10A*\left(P-R-ET\right)$ *Q_wr_* is the total amount of water conservation (m^3^/a), *P* is the rainfall (mm/a), *R* is the surface runoff (mm/a), *ET* is the evapotranspiration (mm/a), and *A* is the area of the ecosystem (ha)	Monetary value of water conservation	Method of market value [29,54,57,58,59] $V_{wr}=Q_{wr}*C_{we}$ *V_wr_* is the monetary value of water conservation (CNY/a), *C_we_* is the market price of domestic water (CNY/m^3^)
	carbon sequestration	Amount of carbon sequestration	Model of oxygen release mechanism [29,54,57,58] $Q_{tCO_{2}}=M_{CO_{2}}/M_{C}*\left(FCS+WCS+GSCS+CSCS\right)$ $Q_{tCO_{2}}\;\mathrm{is}\;\mathrm{the}\;\mathrm{amount}\;\mathrm{of}\;\mathrm{carbon}\;\mathrm{sequestration}\;({\mathrm{tCO}}_{2}/a),\;\mathrm{FCS},\;\mathrm{WCS},\;\mathrm{GSCS},\;\mathrm{CSC}\;\mathrm{are}\;\mathrm{the}\;\mathrm{amount}\;\mathrm{of}\;\mathrm{carbon}\;\mathrm{sequestration}\;\mathrm{in}\;\mathrm{forests},\;\mathrm{wetlands},\;\mathrm{grasslands},\;\mathrm{cultivated}\;\mathrm{lands},\;\mathrm{respectively}\;(\mathrm{tC}/a),\;M_{CO_{2}}/M_{C}=$44/12 is transformation coefficient of molecular weight from CO_2_ to *C*	Monetary value of carbon sequestration	Method of market value [57,58] $V_{Cf}=Q_{CO_{2}}*C_{C}$ *V_Cf_* is the monetary value of carbon sequestration (CNY/a), and *C_C_* is the carbon price (CNY/t)
	oxygen release	Amount of oxygen release	Model of carbon sequestration mechanism [54,57,58] $Q_{OP}=M_{O_{2}}/M_{CO_{2}}*Q_{CO_{2}}$ $Q_{OP}\;\mathrm{is}\;\mathrm{the}\;\mathrm{total}\;\mathrm{amount}\;\mathrm{of}\;\mathrm{oxygen}\;\mathrm{released}\;({\mathrm{tO}}_{2}/a),\;M_{O_{2}}/M_{CO_{2}}$= 32/44 is the coefficient of CO_2_ conversion to O_2_	Monetary value of oxygen release	Method of alternative cost [54,57,58] $V_{OP}=Q_{OP}*C_{O_{2}}$$\;V_{OP}\;\mathrm{is}\;\mathrm{the}\;\mathrm{monetary}\;\mathrm{value}\;\mathrm{of}\;\mathrm{oxygen}\;\mathrm{release}\;(\mathrm{CNY}/a),\;\mathrm{and}\;C_{O_{2}}$ is the oxygen production price (CNY/t)
	flood mitigation	Amount of flood mitigation	Method of water storage [28,54,57,58] $C_{fm}=C_{vfm}+C_{rfm}+C_{lfm}+C_{mfm}$ *C_fm_* is the total amount of flood mitigation (m^3^/a), *C_vfm_*, *C_rfm_*, *C_lfm_*, and *C_mfm_* are the amount of flood mitigation in vegetation, wetlands, lakes, and reservoirs, respectively.	Monetary value of flood mitigation	Method of shadow engineering [54,57,59] $V_{fm}=C_{fm}*P_{w}$ *V_fm_* is the monetary value of flood mitigation (CNY/a), *P_w_* is the engineering cost of the unit storage capacity of the reservoir (CNY/m^3^)
	air purification	Amount of air purification	Model of pollutant purification [57,58] $Q_{ap}=\overset{m}{\underset{i=1}{\sum }}\overset{n}{\underset{j=1}{\sum }}W_{ij}*A_{i}$ *Q_ap_* is the total amount of air purification (kg/a), *W_ij_* is the purification amount per unit area (kg/km^2^/a) of air pollutant *j* of ecosystem *i*, and A_i_ is the area of ecosystem *i* (km^2^)	Monetary value of air purification	Method of market value [57,58] $V_{a}=\overset{n}{\underset{i=1}{\sum }}Q_{api}*C_{i}$ *V_a_* is the monetary value of air purification (CNY/a), *Q_api_* is the purification volume of air pollutant *i* (t/a), *C_i_* is the treatment cost of air pollutant *i* (CNY/t)
	climate regulation	Energy consumption of vegetation transpiration	Evapotranspiration model [54,57,58,59] $E_{tt}=E_{pt}+E_{we}$ $E_{pt}=\overset{n}{\underset{i}{\sum }}EPP_{i}*S_{i}*D*10^{6}/\left(3600*r\right)$ $E_{we}=E_{w}*q*\varepsilon *10^{3}/\left(3600*r\right)+E_{w}*y*\mu$ *E_tt_* is the total energy consumed by transpiration and evaporation (kWh/a), *E_pt_* is the energy consumed by vegetation transpiration, *E_we_* is the energy consumed by evaporation from wetlands, *EPPi* is the heat consumed by transpiration unit area of ecosystem *i* (kJ/m^2^/d). *S_i_* is the area of ecosystem *i* (km^2^), *D* is the number of days with maximum daily temperature over 26 °C, *ε* is the air-conditioning operating coefficient, *r* is the air-conditioning energy efficiency ratio, *E_w_* is evaporation amount (m^3^), *q* is the latent heat of volatilization, *y* is the power consumption of the humidifier to convert 1 m^3^ of water into steam (kWh), *μ* is the operation coefficient of the humidifier	Monetary value of regulating temperature and humidity	Method of alternative cost [54,58] $V_{tt}=E_{tt}*P_{e}$ *V_tt_* is the monetary value of climate regulation (CNY/a), *P_e_* is the local electricity price (CNY/kWh)
		Energy consumption of water surface evaporation			
	water purification	Amount of water purification	Model of pollutant purification [57,58,59] $Q_{wp}=\overset{m}{\underset{i=1}{\sum }}\overset{n}{\underset{j=1}{\sum }}W_{ij}*A_{i}$ *Q_wp_* is the total amount of water purification (kg/a), *W_ij_* is the purification amount per unit area (kg/km^2^/a) of water pollutant *j* of ecosystem *i*, and *A_i_* is the area (km^2^) of ecosystem *i*	Monetary value of water purification	Method of alternative cost [57,58,59] $V_{a}=\overset{n}{\underset{i=1}{\sum }}Q_{api}*C_{i}$ *V_w_* is the monetary value of water purification (CNY/a), *Q_wpi_* is the purification volume of water pollutant *i* (kg/a), *C_i_* is the equivalent tax of water pollutant *i*
	windbreak and sand fixation	Amount of sand fixation	Revised Wind Erosion Equation (RWEQ) [58,61] $G=S_{LP}-S_{LA}\cdot S_{LP}=\left(2Z/S_{P}^{2}\right)Q_{maxP}e^{-{\left(z/s\right)}^{2}}$ $S_{P}=150.71*{\left(WF*EF*SCF*K^{\prime }\right)}^{-0.3711}$ $Q_{maxP}=109.8*\left(WF*EF*SCF*K^{\prime }\right)$ $S_{LA}=\left(2z/S^{2}\right)Q_{max}e^{-{\left(z/s\right)}^{2}}$ $S=150.71*{\left(WF*EF*SCF*K^{\prime }*C\right)}^{-0.3711}$ $Q_{max}=109.8*\left(WF*EF*SCF*K^{\prime }*C\right)$ *G* is the amount of sand fixation (kg/m^2^/a), *S_LP_* is the amount of soil wind erosion under bare soil conditions (kg/m^2^/a), *S_LA_* is the actual amount of soil wind erosion with vegetation coverage (kg/m^2^/a), *Q_max_* is the maximum sand transport capacity of wind (kg/m), *S* is the length of the key block (m), *Z* is the maximum wind erosion distance in the downwind direction (m), *WF* is the weather factor, *EF* is the soil erosion factor, SCF is the soil erodibility, *K*’ is the surface roughness factor, *C* is the vegetation cover factor	Costs of grassland restoration	Method of recovery cost [58] $V_{sf}=\frac{G}{\rho *h}*c$ *V_sf_* is the monetary value of windbreak and sand fixation (CNY/a), *ρ* is the soil bulk density (t/m^3^), *h* is the thickness of soil sand covered by soil desertification (m), *c* is the unit vegetation restoration cost (CNY/m^2^)
	soil erosion prevention	Amount of soil erosion prevention	The revised universal soil loss equation (RUSLE) [29,54,57,58] $Q_{sr}=R*K*L*S*\left(1-C*P\right)$ *Q_sr_* is the total amount of soil erosion prevention (t/a), *R* is rainfall erosivity factor, *K* is soil erodibility factor, *L* is slope length factor, *S* is slope factor, *C* is vegetation cover and management factor, *P* is soil and water conservation measure factor	Monetary value of sedimentation reduction and Monetary value of soil nutrient retention	Method of alternative cost [29,57,58] $v_{\mathrm{sd}}=\lambda *\left(Q_{sr}/\rho \right)*c$ $V_{dpd}=\overset{n}{\underset{i=1}{\sum }}Q_{sr}*C_{i}*P_{i}$ *V_sd_* and *V_dpd_* are, respectively, the monetary value of sedimentation reduction and soil nutrient retention (CNY/a), *c* is the cost of reservoir dredging (CNY/m^2^), *λ* is the sedimentation coefficient, *C_i_* is the pure content of nutrients (%), and *P_i_* is the purchase cost of nitrogen and phosphorus
	biodiversity maintenance	Amount of rare and endangered species	Method of Shannon–Weiner index [58] $G_{bio}=A*\left(1+0.1*\overset{x}{\underset{m=1}{\sum }}E_{m}+0.1*\overset{y}{\underset{n=1}{\sum }}B_{n}+0.1*\overset{z}{\underset{r=1}{\sum }}O_{r}\right)$ *G_bio_* is the total amount of biodiversity maintenance, *E_m_* is the endangerment score of species *m*, *B_n_* is the endemism value of species *n*, *O_r_* is the paleotree age index of species *r*, *A* is the region area (ha)	Monetary value of recreation and experience	Method of conservation Value [58] $V_{bio}=G_{bio}*P_{s}$ *V_bio_* is the monetary value of biodiversity maintenance (CNY/a), *P_s_* is the conservation value of species per unit area (CNY/ha/a)
Cultural services	ecotourism	Number of leisure tourists Or	① Method of statistical survey [57] $N_{t}=\overset{n}{\underset{i=1}{\sum }}N_{i}$ *N_t_* is the total number of leisure tourism, *N_i_* is the number of tourists in tourism area *i* Or	Monetary value of biodiversity maintenance	① Method of travel cost [57,62] $V_{r}=\overset{I}{\underset{i=1}{\sum }}N_{j}*TC_{j}$ ${\mathrm{TC}}_{j}=T_{j}*W_{j}+C_{j}$ $C_{j}=C_{tc,j}+C_{lf,j}+C_{ef,j}$ *V_r_* is the value of cultural services (CNY/a), *TC_j_* is the cost of travel per tourist in region *j*, *T_j_* is the average travel time, *W_j_* is the average salary, *C_j_* is the average direct tourism cost.
		Equivalent of cultural landscape	② Method of equivalent factor [27,30] $E_{C}=\overset{n}{\underset{i=1}{\sum }}X_{i}*A_{i}$ *E_C_* is the equivalent of cultural tourism services, *X_i_* is the equivalent factor of cultural tourism services of ecosystem *i*, *A_i_* is the area of ecosystem *i* (ha)	Equivalent value of cultural landscape	② Method of equivalent factor [27,30,60] $V_{C}=E_{C}*E$ *V_c_* is the monetary value of cultural services (CNY/a), *E* is the economic value of the food production service provided by the modified farmland ecosystem per unit area.

### 2.3. Case Selection and Data Sources

#### 2.3.1. Study Area

**Figure 2:**
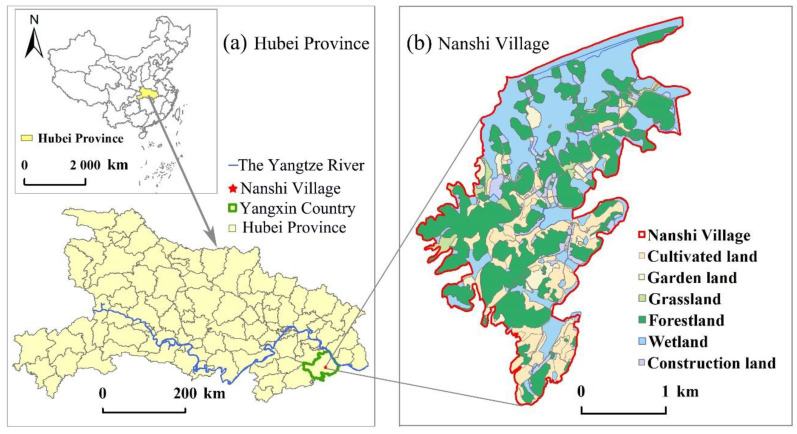
Spatial distribution of the study area.

Promoting the practical exploration of ecological products’ value realization and playing the demonstration role of typical cases is an important initiative to accelerate the establishment of a sound mechanism for realizing the value of ecological products in China. The research explores the path of ecological products’ value realization in Nanshi Village as a case (Figure 2). From the natural environment, Nanshi Village is located in Yangxin County, Hubei Province, China, on the south bank of Fuhe River, a first-class tributary of the Yangtze River. It is within the ecological protection red line of water conservation in the Mufu Mountain region of southeast Hubei Province, with special ecological functions. From the perspective of the human environment, the area of the village is about 6.19 km^2^. In 2020, there were 247 households in the village, with a registered resident population of 1345. The per capita disposable income of the villagers was CNY 13,000. The economic sources were mainly traditional agricultural planting, fishery farming, and migrant workers, which used to belong to the “Mufu Mountain contiguous poverty-stricken area.”

Nanshi Village is rich in ecological resources and backward in ecological economics, which has the general characteristics of rural development in China. The practice of utilizing the comparative advantages of the rural ecological-environmental to achieve social-economic goals is not only of great significance to its growth but also has general reference value for the endogenous development of other similar villages. That is why we chose it as a case to study how to transform ecological-environmental advantages into the benefits of social-economic growth.

#### 2.3.2. Data Sources

The data required for the research mainly include geospatial data, monitoring data, and statistical data. Among them, the geospatial data (basic geographic data, such as administrative boundaries, roads, and land use) were obtained from Yangxin County Natural Resources Bureau and Hubei Provincial for Common GeoSpatial Information Services (https://hubei.tianditu.gov.cn/, accessed on 1 September 2021). The slope and slope length factors were calculated according to “ASTER GDEM 30 m resolution digital elevation data” (http://www.gscloud.cn/, accessed on 1 September 2021), and “Normalized Vegetation Index NDVI 30 m raster data” were obtained from the Geographic Information Monitoring Cloud Platform (http://www.dsac.cn/, accessed on 1 September 2021). The monitoring data (meteorological data, such as rainfall and evapotranspiration, soil factor data such as organic matter content, air pollutant purification data such as degradable particulate matter, water pollutant concentration data, such as BOD, COD, and total nitrogen, and species conservation data, such as old and famous trees) were obtained from Yangxin Meteorological Bureau, Soil and Fertilizer Station, Ecological Environment Bureau, Water Resources Bureau, Forestry Bureau, and other related departments. Statistical data (such as agriculture, forestry, animal husbandry, and fishery products) were obtained from the village accounts and interviews with village cadres. Regional social-economic data were obtained from the Yangxin County Bureau of Statistics and China Statistical Yearbook.

## 3. Results

### 3.1. Value and Structure Characteristics of Ecological Products in Nanshi Village

#### 3.1.1. Calculate the Value of Ecological Products

According to Section 2.1, the value of ecosystem services includes three major types: provisioning services, regulating services, and cultural services. According to the calculation methods in Section 2.2 and Table 3, combined with the actual situation of Nanshi Village, the value of different ecosystem services was calculated (Table 4).

(1) Provisioning services. This type includes five items of agricultural crop, forestry, animal husbandry, fishery, and other production. In 2020, the total area of cultivated land in Nanshi Village was 0.95 km^2^, the biophysical quantity of agricultural crop products was 558.7 t, and the monetary value was CNY 308.16 × 10^4^. The total area of forest land in Nanshi Village was 2.51 km^2^, with a monetary value of CNY 102.33 × 10^4^. The animal husbandry operation includes two kinds of livestock poultry breeding and egg production, and the sum of the economic value was CNY 95.23 × 10^4^. The wetland area of Nanshi Village was 1.68 km^2^, the output of freshwater aquaculture products was 300 t, and the monetary value was CNY 450 × 10^4^. The supply of other products, such as ecological energy, was tiny in Nanshi Village and thus was not included in the accounting for the time being. In 2020, the total monetary value of provisioning services in Nanshi Village was CNY 955.72 × 10^4^.

(2) Regulating services. This type includes ten items: Water connotation, flood mitigation, carbon sequestration, oxygen release, windbreak and sand fixation, soil erosion prevention, climate regulation, air purification, water purification, and biodiversity maintenance. In 2020, the annual water connotation amount of Nanshi Village was 537.19 × 104 m^3^, with a monetary value of CNY 1557.84 × 10^4^. The amount of flood mitigation was 83.85 × 104 m^3^, with an economic value of CNY 668.15 × 10^4^. The sequestrated amount of carbon was 2534 t, and the monetary value was CNY 304.08 × 10^4^. The amount of oxygen released was 1842.88 t, and the economic value was CNY 248.79 × 10^4^. The total amount of sand fixation was 44,695.56 t, and the monetary value was CNY 132.92 × 10^4^. The amount of soil erosion prevention was 112,407 t, of which the economic value of reducing sedimentation was CNY 37.72 × 10^4^, the value of soil nutrient retention was CNY 653.04 × 10^4^, and the total monetary value was CNY 690.76 × 10^4^. The total energy consumed by transpiration and evaporation was 18,122,998 kWh, and the economic value was CNY 1011.26 × 10^4^. The total amount of air purification for SO_2_, fluoride, nitrogen oxides, and floating and sinking particles was 385.82 t, and the total monetary value was CNY54.10 × 10^4^. The total amount of water purification was 60,342 kg, and the total economic value was CNY 14.62 × 10^4^. The diversity index was 1.7, the total species conservation was 426.39, and the monetary value of biodiversity maintenance was CNY 213.19 × 10^4^. In 2020, the total monetary value of ecological regulation services in Nanshi Village was CNY 489.571 × 10^4^.

(3) Cultural services. Ecosystem cultural services mainly correspond to the contribution of nature to ecotourism. The ecosystems of mountains, rivers, forests, farmlands, lakes, and grasslands all have potential values of cultural services, but the village is limited by the development stage, development background, or development orientation. The marketization of cultural services’ value is insufficient, and it is not easy to quantify its value directly. Therefore, the “modified equivalent factor method” was used to calculate the potential value: The grain production value, spatial heterogeneity coefficient, resource scarcity coefficient, and social-economic adjustment coefficient were used for correction [30,60], and the value equivalent of Yangxin County was estimated as 1876.54 CNY/ha in 2020. The value coefficients of various ecosystems were calculated with reference to “The equivalent coefficients table for ecosystem service value (ESV) per unit area for the ecosystems and ecosystem services” of Xie et al. [57]. He and his team proposed an integrated method for the dynamic evaluation of Chinese terrestrial ecosystem service value based on Costanza’s method [27]. It can be calculated that the total monetary value of cultural tourism services in Nanshi Village in 2020 was CNY 114.96 × 10^4^.

Accordingly, the total potential GEP of Nanshi Village in 2020 was CNY 5966.39 × 10^4^, and the per capita GEP was as high as CNY 40588, three times the per capita disposable income of rural permanent residents in Yangxin County in the same period. Promoting the actual transformation of the potential value of ecological products is of great significance for regional development.

#### 3.1.2. Analysis of Ecological Value Structure

The key to realizing the value of ecological products lies in exerting the comparative advantage of ecosystem services. Clarifying the value structure of ecological products is conducive to identifying the comparative advantage of ecosystem services.

Based on the ecosystem functions perspective. Overall, the potential value of regulating services was CNY 4895.71 × 10^4^, with the highest contribution rate of 82.05% to GEP. The potential value of provisioning services was CNY 955.72 × 10^4^, with a contribution rate of 16.02%. The potential value of cultural services was CNY 114.96 × 10^4^, with the lowest contribution rate of 1.93%. Specifically, the potential value of ecosystem functions, in descending order, was as follows: Water connotation, climate regulation, soil erosion prevention, flood mitigation, fishery production, agricultural crop production, carbon sequestration, oxygen release, biodiversity maintenance, windbreak and sand fixation, the contribution of nature to ecotourism, forestry production, animal husbandry production, air purification, and water purification. Ecological regulating service is the core ecosystem function of Nanshi Village. The realization of ecological products’ value focuses on promoting the transformation of the potential value of regulating services function with water connotation, climate regulation, soil erosion prevention, flood mitigation, and carbon sequestration, and oxygen release as the core.

Based on the perspective of ecological resource types. According to the actual natural resource assets of Nanshi Village, the ecosystems were divided into five resource types: Cultivated land, forest land, garden land, grassland, and wetland. The potential value of various types of natural resources and the contribution rate to GEP in descending order are forestland resources (CNY 2530.98 × 10^4^, 42.42%), wetland resources (CNY 2152.76 × 10^4^, 36.08%), cultivated land resources (CNY 993.61 × 10^4^, 16.65%), garden resources (CNY 196.66 × 10^4^, 3.3%), and grassland resources (CNY 92.40 × 10^4^, 1.55%). Forests, wetlands, and cultivated land resources constitute a significant barrier to the ecological protection of Nanshi Village and are the leading spatial carriers for realizing ecological values in Nanshi Village.

Based on the superposition analysis of ecosystem functions and ecological resource types. In general, 47.74% of the potential value of regulating services was contributed by forestland resources and 32.98% by wetland resources. Moreover, 50.06% of the potential value of product supply services was contributed by wetland resources and 34.14% by cultivated land resources. Furthermore, 51.59% of the potential value of cultural services was contributed by wetland resources and 48.67% by forestland resources. Specifically, cultivated land resources had the highest contribution rate to agricultural crop production. Forestland resources had the highest contribution rate in nine categories of ecological service functions: Forestry production, animal husbandry production, water connotation, carbon sequestration, oxygen release, air purification, windbreak and sand fixation, soil erosion prevention, and biodiversity maintenance. Wetland resources had the highest contribution rate among the five categories of ecosystem service functions: Fishery production, flood mitigation, climate regulation, water purification, and contribution of nature to ecotourism. Based on maintaining the advantages of provisioning services of cultivated land and wetland resources. Nanshi Village should focus on promoting the indirect transformation of the potential value of the comparative advantage of regulating services with forest land and wetland resources as the carrier and relying on wetland and forest land resources to promote the comprehensive realization of the potential value of cultural services.

### 3.2. Model Selection and Path Exploration of Rural Revitalization in Nanshi Village

#### 3.2.1. Model Selection of Rural Revitalization

According to Section 2.1, the matching relationship between the division of major function zones and the classification of ecological services is the scientific basis for the selection of rural revitalization models. However, the realistic development conditions are factors that cannot be ignored in determining rural revitalization models.

1. The scientific basis for choosing the rural revitalization model should be followed. According to the “Major Function Oriented Zoning Planning of Hubei Province”, Yangxin County, where Nanshi Village is located, belongs to the main agricultural production zones, and the primary function of the region is to provide agricultural products. The value structure analysis indicated that the contribution rate of regulating services had the highest contribution rate to the GEP of Nanshi Village, and the ecological regulation service was the local dominant advantage. In our study, by matching the major function zoning and ecological services classification, we find that Nanshi Village was an uncoordinated type (main agricultural production zone-regulating services dominant type). According to Section 2.1 and Table 1, theoretically, the rural revitalization of Nanshi Village chooses the compatible “industrial ecologization-ecological productization” model. In reality, ecological equity transactions require a certain amount of scale. The scale limits the vast scattered villages represented by Nanshi Village. Because the market mechanism for trading ecological rights and interests has not yet been established and perfected, it is still impossible to realize the ecological values through the “ecological productization” model. Therefore, at present, on the premise of strictly adhering to the red line of arable land protection and ensuring that the value of agricultural products and services is not reduced, “industrial ecologization” should be taken as the leading model.

2. The realistic conditions for choosing the rural revitalization model should be considered. The analysis focused on the existing industrial base and the willingness of villagers to develop. The village is adjacent to the Fuhe River in the north and the East-West Lake in the east, with beautiful natural scenery. It is the birthplace of “Yangxin Fishing Song”, which is an integral part of the river and lake culture in the Yangtze River Basin and has a profound cultural heritage. It is located in the suburbs of Yangxin County, with a superior geographical location. The superposition of natural, cultural, and regional advantages has successively attracted leading enterprises such as the Zhexing Agricultural Ecotourism Development Company and the Hubei Fengwa Tourism Management Company to settle in villages for investment and development. The village has an excellent realistic foundation for developing ecological tourism industry. Through three industrial surveys and field interviews, we have fully understood the demands of villagers’ representatives, village cadres, heads of cooperatives, and other groups for industrial development, which were highlighted by their great enthusiasm and high expectations for the development of rural tourism. The development of the tourism industry not only brings farmers direct income from the transfer of land management rights but also provides indirect income from the employment of labor in scenic spots. Farmers also desire the development opportunities of catering, accommodation, and agricultural product sales that the spillover of tourism benefits may bring. With the superposition of the real industrial foundation and the villagers’ development willingness, in the actual development of Nanshi Village, the “ecological industrialization” model should be used as a supplement.

Accordingly, the endogenous development model of rural revitalization in Nanshi Village is determined by “industrial ecologicalization as the leading model and ecological industrialization as the supplement model”.

#### 3.2.2. Path Exploration of Rural Revitalization

According to Section 2.1 and Section 3.2.1, the development path corresponding to the endogenous development model of rural revitalization, which is “industrial ecologicalization as the leading model and ecological industrialization as the supplement model”, is “selling products” and “selling landscape services”. Accordingly, the ecological industrial system of “forestry and fishery leading, agriculture and tourism integration” will be constructed, and the leading section selected, and the specific related industries matched according to the economic and technological connection of the industry. 

1. Take “industrial ecologicalization” as the leading model.

Relying on valuable ecological resources such as woodlands and wetlands, and with local farmers as the main body of development, we will deploy leading ecological forestry and fishery industries. It will realize the high-quality manifestation and high-value transformation of the potential value of forest land and wetland resource provisioning services and regulating services in the form of “selling products” and ecological or brand value-added. 

a. For the ecological forestry industry. The national policy opportunity of “promoting the high-quality development of woody grains and oils and under-forest economy” should be explored and combined with Yangxin County’s development plan of building a high-quality demonstration garden around “fruit, tea, medicine, mulberry, mushroom, and hemp”. It is also necessary to focus on the production conditions of forest land resources in Nanshi Village and the realistic basis for developing Moso bamboo, Camellia oleifera, and Citrus industries. It uses more than 2.47 km^2^ of high-quality forest land resources as the carrier, taking “forest medicine”, “forest fruit”, and “forest birds” as the direction to carry out three-dimensional planting and breeding of ecological forestry. It will drive the development of Moso bamboo collection and processing and traditional Chinese medicine materials processing and packaging. The above industries are designed with the expectation that manifests the ecological regulating service value of soil erosion prevention and water connotation of forestland resources in the form of ecological products or brand value-added. 

b. For the ecological fishery industry. On the one hand, a stand on the national policy orientation of “high quality and green development of ecological fishery on large water surface” should be taken and focus should be given on the development plan of “building a brand of geographical trademarks of organic fishery” in Yangxin County. On the other hand, the government should take advantage of the rich ecological resources of the wetlands, the mature ecological fishery farming technology, and the stable sales channels of Nanshi Village. Taking more than 1.53 km^2^ of high-quality wetland resources as the carrier, it continues to consolidate and expand the leading industry of ecological fishery breeding and combines the traditional production of local smoked fish. It will drive the processing and sales of ecological fishery products and improve the market competitiveness of products, as well as increase the ecological or brand premium of products. The ecological regulating services value of water quality purification of wetland resources will be revealed.

2. Take “ecological industrialization” as the supplement model.

The ecotourism industry should be planned scientifically. The investment opportunity of the “Nanshi Fishing Song Ecological Cultural Tourism” project in Nanshi Village should be taken. Moreover, relying on diversified social investment and management entities and carrying out the industrialized development of ecological culture and tourism under the premise of minimizing human disturbance is essential. The purpose is to solve the shortcomings of “talents, capital, technology, and management” in rural areas with the aim of realizing the goal of “attracting people into the countryside” via rural scenery tourism. Through “selling landscape services”, the potential value of regulating services (oxygen release, air purification, water connotation, water purification, and climate regulation) and cultural services value are manifested in the form of the ecological and cultural landscape services. It adheres to the development principle of “utilizing local resources, developing local industries, driving residents’ employment, and realizing residents’ prosperity”. The extension of the ecological culture and tourism chain is driving the development of the leading industry, ecological forestry, and fishery, with local farmers as the primary business entities. On these grounds, giving full play to the advantages of “land, labor, ecology and culture” in rural areas and realizing “selling products” utilizing rural ecological shopping is important. Finally, the total value of provisioning services, regulation services, and cultural services in the form of final products and services should be realized.

3. Shaping the industrial spatial structure of “three zones and five parks”.

**Figure 3:**
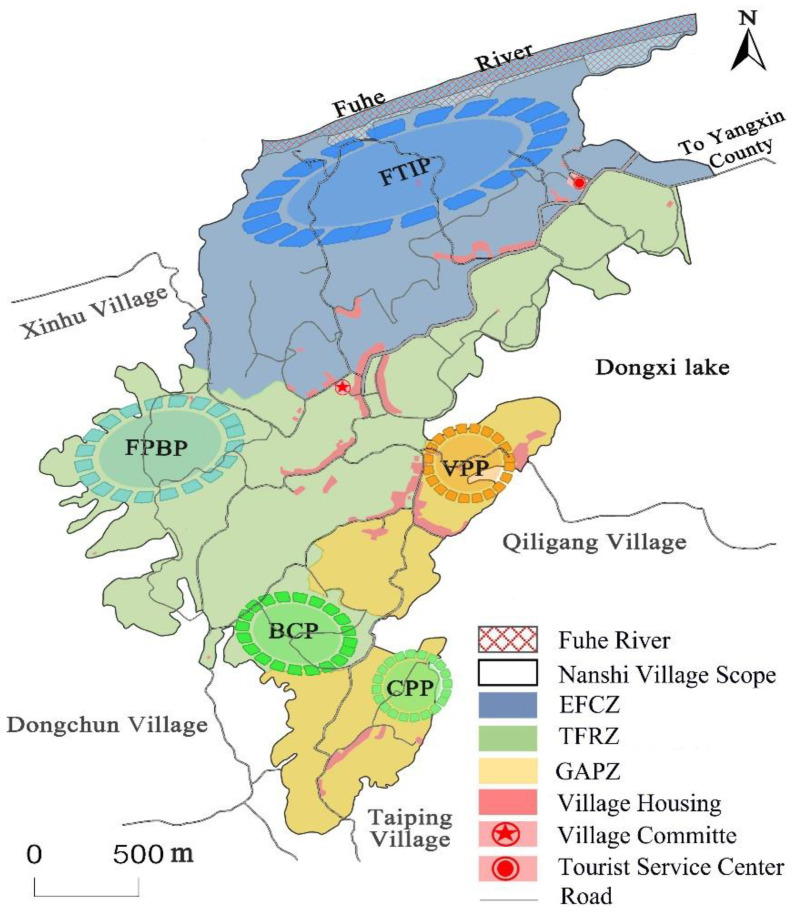
Industrial development space zoning in Nanshi Village.

The linkage and support of industrial layout should be considered, and the advantage of ecosystem services value should be matched scientifically with the industrial spatial development layout (Figure 3). First, using the grid analysis function of ArcGIS software to calculate the value of different types of ecological service functions at the grid scale of 200 × 200 m. Second, the Self-Organizing Feature Mapping (SOFM) was used to cluster and partition the study area according to the principle of “spatial connection and similar attributes” [63]. Specifically, the values in the ArcGIS spatial database were exported and converted into text documents by Surfur software, and the document data and SOFM-related functions provided by the Matlab neural network toolbox were called on the Matlab platform for programming. The main functions include initializing weights, learning, and training, and competitive activation. After the program runs, the results were imported into ArcGIS spatial database. Third, combined with the actual situation of land use and industrial development planning, the GIS spatial overlay analysis function was used to modify the industrial spatial partition. Finally, three major industrial spatial zones are proposed to be constructed, including the Ecological Fishery Cultural Service Realization Zone (EFCZ), the Three-dimensional Forestry Regulating Service Transformation Zone (TFRZ), and the Green Agricultural Provisioning Service Improvement Zone (GAPZ), with the aim of, focusing on the realistic transformation of the multi-functional value of the ecosystem. Specifically, the EFCZ will take the wetland resources north of the village as the spatial carrier, aiming to realize the comprehensive benefits of ecological services. The TFRZ will bring the forest land resources to the middle of the town as the spatial carrier, focusing on learning the value of regulating and provisioning services. The GAPZ will take the cultivated land and garden resources in the central and southern of the village as the spatial carrier, focusing on realizing the value of provisioning and ecological regulating services. According to the results of industrial spatial zoning, synthesizing the existing industrial development status, combined with the villagers’ development willingness, decomposing the tasks of industrial structure planning, and carrying out the construction of industrial parks are necessary. The plan is to build five industrial parks in Nanshi Village, including Fishery and Tourism Integration Park (FTIP), Forest Planting and Breeding Park (FPBP), Bamboo Collection Park (BCP), Vegetable Plantation Park (VPP), and Citrus Planting Park (CPP). Among them, the FTIP supports the development of the EFCZ in the north of the village. The FPBP supports the development of the TFRZ in the middle and western parts of the village. The BCP supports the development of the TFRZ in the middle and southern of the village. The VPP supports the development of the GAPZ in the middle and eastern parts of the village. The CPP is used to support the development of GAPZ in the southern parts of the village. 

Finally, the ecological industry structure planning will be implemented in Nanshi Village, promoting the high-quality development of ecological leading industries and realizing the high-value transformation of ecological products.

## 4. Discussion

Rural areas are rich in ecological resources but often backward in terms of the ecological economy. They have the dual tasks of ecological environment protection and comprehensive rural development. Transforming the advantages of the ecological environment into social and economic growth is of great significance in realizing villages’ sustainable development and protecting people’s well-being in developing countries. This paper was guided by the ecological economy theory of “two mountains”. Our study integrated multi-disciplinary ecology, geography, economics, and planning approaches. It examined the comprehensive proposition of the two-way transformation of “ecological and environmental advantages” and “social and economic development” with integrated disciplinary thinking. We also constructed a theoretical framework to realize the endogenous development of rural areas by giving full play to the comparative advantage of abundant natural resource assets and manifesting the potential value of ecological products. Based on existing research, we constructed indicators and methods for accounting for the value of ecological products suitable for rural areas. Finally, we conducted an empirical study to explore the path of realizing the value of ecological products, taking Nanshi Village, Yangxin County, China, which has the dual characteristics of abundant environmental resources and a backward social economy, as an example. The research has theoretical and practical value.

### 4.1. Theory and Method Innovation

(1) The “two mountains” theory can open up a new perspective for rural revitalization from the standpoint of ecological economy. The essence of rural revival is rural development, and cultivating endogenous power based on its advantages is an effective path to sustainable rural development. The core idea of the “two mountains” theory is that high-quality ecological products can not only meet people’s growing needs for a better environment directly but also meet people’s needs for a better life through ecological value transformation. Rural areas have an excellent natural environment and great ecological products. Under the guidance of the “two mountains” theory, transforming the ecological and environmental advantages into social and economic development advantages can provide a new perspective and general reference for the endogenous development of rural revitalization.

(2) The analysis framework of endogenous development is based on the matching relationship between the division of major function zones and the classification of ecological services, which can provide a theoretical reference for selecting rural revitalization models and paths. Rural revitalization must be compatible with the strategy of MFOZ, and the division of major function zones should clarify the constraints of regional development. Rural revival also should give full play to the local ecological comparative advantages and define the critical points of local development by classifying ecological services. The dominant model and path of rural revitalization should be determined by matching the relationship between the two factors. The study can contribute disciplinary wisdom from a spatial perspective to promoting rural revival in a zonal and categorical manner in the context of strategic transformation of ecological civilization and has universal reference value for developing villages with comparative ecological advantages.

(3) The scientific measurement of ecological product value is the essential support for realizing the value of ecological products. Based on the international guidelines and Chinese national standards and drawing upon the previous research results and Chinese pilot examples, a scientific and relatively complete construction of ecological products value accounting indicators, biophysical quantity, and monetary value accounting methods applicable to Chinese villages is another contribution of this study. In previous studies, the ecosystem services value accounting mostly used the ecosystem services value equivalent table developed by Costanza et al. [27,36]. This method was widely used because of its simplicity of operation but was criticized for ignoring regional differences and being too subjective and because of the relatively low market acceptance of the accounting price. The ecosystem services value calculated based on the biophysical quantity and market value of ecological products in the study area was mostly accounted for separately by taking one or several types of ecosystem services as an example. The comprehensive ecosystem services value research involving the biophysical quantity and monetary value was insufficient, and this paper can be regarded as a valuable supplement.

### 4.2. Policy Recommendations

The case study showed that the benefits provided by ecosystems to the development of human society would be underestimated if it is measured only by the provisioning services. Great potential for the actual transformation of the potential values of regulating services and cultural services exists. Therefore, we propose several general recommendations for the development of a rural ecological economy: Giving full play to the comparative ecological advantages of rural areas, taking the realization of the total value of ecological products (provisioning services, regulating services, and cultural services) as the starting point, promoting the return of modern elements such as “capital, talent, and technology” to rural areas, and activating the reorganization of the stock elements such as “land, labor, and industry” in rural areas. Finally, through the prosperity of the ecological industry (economic structure), driving the farmers to live a rich life (social structure), promoting rural ecological livability (spatial structure), and ultimately realizing the comprehensive development of rural production, life, and ecology. We can also verify the two-way relationship between ecosystem services and people’s well-being in the relevant studies of scholars. The survey of Ciftcioglu et al. [64] confirmed a high correlation between ecosystem provisioning services and the basic materials to maintain a good life. A high positive correlation between cultural services and the development of good social relationships can also be observed. Li et al. [65] also found that ecosystem services in mountain areas and mountain residents’ well-being showed a double increase trend in the research of poor mountainous regions in China. Zhang et al. [66] also considered ecological industrialization to be essential in promoting the virtuous circle of environmental construction and industrial development.

### 4.3. Limitations and Research Prospects

The selection of different types of rural revitalization models and paths under the view of the “two mountains” theory is based on the conditions superposition of the differences in major functional zoning and differences in local ecological comparative advantages. The research sought to provide theoretical support for the practice of promoting rural revitalization by zoning and classification from a spatial perspective, to give a framework and provide an idea. Rural revitalization is a complex project involving two giant systems in urban and rural areas, which urgently need systematic theory support [20,67]. With the complexity of the real world, the choice of rural revitalization models and the exploration of paths are often the results of the combined effect of multiple factors, such as comparative ecological advantages, realistic industrial foundation, national policy orientation, and villagers’ development willingness. In addition, the connotation, evaluation indexes, accounting methods, realization mechanisms of the “two mountains” theory, and ecological product values need to be deepened [29,47,68,69]. In fact, not only do the differences for villages in different combinations of major function divisions and ecological services classifications, but also the same combination, will have regional development differences, and the emphasis on comprehensive rural development in different stages of the same region is different. In China, based on the regional and spatial differences, scholars have explored the selection of rural revitalization models and paths in different types of areas, such as the Hilly-Gully region of the Loess Plateau, the traditional plain rural area of central China, and the Beijing-Tianjin-Hebei metropolitan region from the perspectives of land consolidation, industrial clusters development, urban-rural functional transformation, and urban-rural integration development [70,71,72,73]. Under the guidance of the theoretical framework, the specific selection of the rural revitalization models and paths should be carried out according to local conditions and times.

Under the background of ecological civilization construction and relying on China’s vast territory and long history, the proper direction for academic research is to refine rural revitalization models of different types of zones and scientifically study and judge the paths of rural revitalization in the rich and colorful rural development practices. It is also the direction of the author’s future efforts.

## 5. Conclusions

(1) The value realization of ecological products is a practical path for the endogenous development of rural revitalization. In China, rural areas are vast, and the geographical environment, human activities, and interactions are very different. Local ecological comparative advantage development must be constrained by orientating major regional functions. The matching relationship between the division of major function zones and the classification of ecological services can provide a scientific basis for realizing the value of environmental products and deconstructing the rural revitalization models. There is a corresponding primary relationship between the division of major function zones and the classification of ecological services. The superposition of the two can build a “3 × 3” relationship matrix and get two types and nine categories of matching relationships, corresponding to nine kinds of rural revitalization models and path choices.

(2) In 2020, the potential GEP of Nanshi Village was CNY 5966.39 × 10^4^, and the per capita GEP was three times the per capita disposable income of rural permanent residents in Yangxin County in the same period. It showed that realizing the ecological products’ value will be a beneficial attempt to promote local development with high quality. Among them, the potential value of regulating services was CNY 4895.71 × 10^4^, with the highest contribution ratio to GEP of 82.05%. The potential value of provisioning services was CNY 955.72 × 10^4^, with the contribution ratio to GEP of 16.02%. The potential value of cultural services was CNY 114.96 × 10^4^, with the contribution ratio to GEP of 1.93%. It showed that regulating services was the core of the ecosystem functions of Nanshi Village. In addition, 47.74% of the ecological regulating services were contributed by forest land resources, and wetland resources contributed 32.98%. Relying on the carrier of forest land and wetland resources, manifesting the potential value of ecological regulating services was the key to realizing the value of environmental products in Nanshi Village.

(3) Choosing the development model of “industrial ecologicalization as the leading model and ecological industrialization as the supplement model”, constructing the ecological industrial structure system of “forestry and fishery leading, agriculture and tourism integration,” and shaping the industrial spatial structure of “three zones and five parks”. Through the two paths of “selling products” and “selling landscape services”, on the premise of ensuring that it will not reduce the agricultural products and services, the value of regulating services and cultural services embedded in an excellent ecological environment will be manifested and strengthened in the form of final products and services, which is a viable path for the endogenous development of rural revitalization in Nanshi Village.

## Figures and Tables

**Table 1:** Matrix of the relationship.

Types	Regulating Services Dominant	Provisioning Services Dominant	Cultural Services Dominant
Key ecological function zones	(1,1)	(1,2)	(1,3)
Main agricultural production zones	(2,1)	(2,2)	(2,3)
Urbanized zones	(3,1)	(3,2)	(3,3)

Note: The above table is a “3 × 3” relationship matrix, with 3 rows and 3 columns, of which the former represents the rows, and the latter represents the columns. For example, (1,1) represents the relationship combination of the first row and the first column (key ecological function zones, regulating services dominant).

**Table 2:** Models and implementation paths of rural revitalization.

Types	Matching Relationships	Dominant Models	Main Paths
coordinated types	(1,1)	Ecological productization	Selling equities: Sharing ecological equity trading market, participating in regional coordination of environmental protection
	(2,2)	Industrial ecologization	Selling products: Building an ecological industry system, constructing an ecological product structure
	(3,3)	Ecological industrialization	Selling landscape services: Giving play to the advantages of ecological environment and developing rural eco-tourism
uncoordinated types	(1,2)	Ecological productization- Industrial ecologization	Constrained by the realization of ecological functions, “Selling equities” and “Selling products”
	(1,3)	Ecological productization- Ecological industrialization	Constrained by the realization of ecological functions, “Selling equities” and “Selling landscape services”
	(2,1)	Industrial ecologization- Ecological productization	With the guarantee of agricultural production as the constraint, “Selling products” and “Selling equities”
	(2,3)	Industrial ecologization- Ecological industrialization	With the guarantee of agricultural production as the constraint, “Selling products” and “Selling landscape services”
	(3,1)	Ecological industrialization-Ecological productization	To promote the development of aggregation as a constraint, “Selling landscape services” and “Selling equities”
	(3,2)	Ecological industrialization- Industrial ecologization	To promote the development of aggregation as a constraint, “Selling landscape services” and “Selling products”

**Table 4:** GEP and the percent of each ecosystem service item in the grand total monetary value of Nanshi Village in 2020.

Types of Ecosystem Service	Accounting Items	Total Monetary Value (10^4^ CNY)	Percent of Total Value, %	Monetary Value of Cultivated Land (10^4^ CNY)	Percent of Total Value, %	Monetary Value of Forestland (10^4^ CNY)	Percent of Total Value, %	Monetary Value of Garden (10^4^ CNY)	Percent of Total Value, %	Monetary Value of Grassland (10^4^ CNY)	Percent of Total Value, %	Monetary Value of Wetland (10^4^ CNY)	Percent of Total Value, %
Provisioning services	agricultural crop products	308.16	5.16	308.16	5.16	—	—	—	—	—	—	—	—
	forestry products	102.33	1.72	—	—	102.33	1.72	—	—	—	—	—	—
	animal husbandry products	95.23	1.6	18.11	0.30	42.55	0.71	3.85	0.06	2.28	0.04	28.45	0.48
	fishery products	450	7.54	—	—	—	—	—	—	—	—	450	7.54
Total provisioning services		955.72	16.02	326.27	5.47	144.88	2.43	3.85	0.06	2.28	0.04	478.45	8.02
Regulating services	water connotation	1557.84	26.11	387.86	6.50	1022.71	17.14	92.43	1.55	54.84	0.92	—	—
	carbon sequestration	304.08	5.1	31.92	0.53	228.88	3.84	20.94	0.35	0.12	0.00	22.21	0.37
	oxygen release	248.79	4.17	26.12	0.44	187.27	3.14	17.14	0.29	0.1	0.00	18.17	0.30
	air purification	54.1	0.91	8.98	0.15	30.58	0.51	2.07	0.03	0.99	0.02	11.47	0.19
	flood mitigation	668.15	11.2	—	—	—	—	—	—	—	—	668.15	11.20
	climate regulation	1011.26	16.95	6.18	0.10	114.05	1.91	8.43	0.14	2.65	0.04	879.96	14.75
	water purification	14.62	0.25	—	—	—	—	—	—	—	—	14.62	0.25
	windbreak and sand fixation	132.92	2.23	33.09	0.55	87.26	1.46	7.89	0.13	4.68	0.08	—	—
	soil erosion prevention	690.76	11.58	171.98	2.88	453.48	7.60	40.98	0.69	24.32	0.41	—	—
	biodiversity maintenance	213.19	3.57	—	—	213.19	3.57	—	—	—	—	—	—
Total regulating services		4895.71	82.05	666.13	11.16	2337.42	39.18	189.88	3.18	87.7	1.47	1614.58	27.06
Cultural services	ecotourism	114.96	1.93	1.21	0.02	48.67	0.82	2.93	0.09	2.42	0.04	59.72	1.00
Grand Total		5966.39	100	993.61	16.65	2530.976	42.42	196.66	3.30	92.4	1.55	2152.76	36.08

Note: The “Total provisioning services” includes four specific accounting items for the type of provisioning services; the “Total regulating services” includes ten specific accounting items for the type of regulating services; the “Grand Total” includes all the specific accounting items. “Percent of total value”, where the total value corresponds to the total potential GEP, which is CNY 5966.39 × 10^4^.

## Data Availability

Not applicable.
